# Supplementary material for: Content-rich biological network constructed by mining PubMed abstracts
Source: BMC Bioinformatics. 2004 Oct 8;5:147. doi: 10.1186/1471-2105-5-147 (PMC528731; doi:10.1186/1471-2105-5-147)
Supplement: Additional File 5 — The original Chilibot query results of the term "long-term potentiation (LTP)" and 22 other terms, limiting the latest references analyzed to the years 1990, 1995, 2000, and 2004. [file 1471-2105-5-147-S5.bz2 › chilibotAdditionalFile5/ltp2004/html/LTP_ACTIN.html]

 


 **LTP** and **ACTIN** 
  
Found 36 abstracts in PubMed,  **30 abstracts were retrieved and analyzed**.  


---

 Search Google  |
 PDF files only 
|  EDU domain only 

---

**Interactive relationship** (e.g. stimulation, inhibition, etc)

- New results show that  **LTP**  produces an increase in  **actin**  rich spines.  Ref: 12741982 Neuron, 2003
- F  **actin**  is required for  **LTP**  induction, but the underlying mechanisms are unclear.  Ref: 12741982 Neuron, 2003
- Hippocampal  **LTP**  is accompanied by enhanced F  **actin**  content within the dendritic spine that is essential for late  **LTP**  maintenance in vivo.  Ref: 12741991 Neuron, 2003
- Here we used an in vivo dentate gyrus  **LTP**  model to show that  **LTP**  induction is associated with  **actin**  cytoskeletal reorganization characterized by a long lasting increase in F  **actin**  content within dendritic spines.  Ref: 12741991 Neuron, 2003
- These results suggest that postsynaptic  **actin**  filaments are involved in a dynamic process required to maintain AMPAR mediated transmission and to enhance it during  **LTP** .  Ref: 10341235 J Neurosci, 1999
- These observations suggest that mechanisms regulating the spine  **actin**  cytoskeleton contribute to the persistence of  **LTP** .  Ref: 12741991 Neuron, 2003
- Dynamic  **actin**  filaments are required for stable long term potentiation  [ **LTP** ]   **LTP**  in area CA1 of the hippocampus.  Ref: 10823894 Proc Natl Acad Sci U S A, 2000
- There is evidence that reorganization of the neuronal  **actin**  cytoskeleton is important for consolidation of long term potentiation  [ **LTP** ] .  Ref: 11823064 Neuroscience, 2002
- The results are consistent with a model in which dynamic  **actin**  filaments play an essential role in the molecular mechanisms underlying the early maintenance phase of  **LTP** , such as growth of new synaptic connections or conversion of silent synapses.  Ref: 10823894 Proc Natl Acad Sci U S A, 2000
- **Actin**  filaments control cell morphology and are essential to the growth of dendritic spines and the plasticity of hippocampal long term potentiation  [ **LTP** ]   **LTP** .  Ref: 12799133 Mol Cell Neurosci, 2003

**Parallel relationship** (e.g. studied together, co-existance, homology, etc.)

- Regulated expression of an  **actin**  associated protein, synaptopodin, during long term potentiation  [ **LTP** ] .  Ref: 11595771 J Neurochem, 2001
- **Actin**  in synaptic cytoskeleton during long term potentiation  [ **LTP** ]  in hippocampal slices.  Ref: 1811261 Acta Histochem Suppl, 1991
- Using a combination of genetic manipulations and electrophysiological recordings in mice, we have demonstrated that LIMK 1 signaling is important in vivo in the regulation of the  **actin**  cytoskeleton, spine morphology, and synaptic function, including hippocampal long term potentiation  [ **LTP** ]   **LTP** , a prominent form of long lasting synaptic plasticity thought to be critical to memory formation.  Ref: 14513866 Rev Neurosci, 2003
- Inhibition of  **actin**  polymerization with latrunculin A impaired late phase of  **LTP**  without affecting the initial amplitude and early maintenance of  **LTP** .  Ref: 12741991 Neuron, 2003
- A role of  **actin**  filament in synaptic transmission and long term potentiation  [ **LTP** ] .  Ref: 10341235 J Neurosci, 1999
- Our findings provide evidence for a novel biochemical pathway involving phosphoinositide 3 kinase and p70 S6 kinase that is important for the maintenance of hippocampal long term potentiation  [ **LTP** ] , possibly via regulation of  **actin**  dynamics.  Ref: 11823064 Neuroscience, 2002
- **Actin**  s actions in  **LTP**  induced synapse growth.  Ref: 12741982 Neuron, 2003
